# Thrombocytopenia in the Setting of Hemodialysis Using Biocompatible Membranes

**DOI:** 10.1155/2012/358024

**Published:** 2012-10-09

**Authors:** Kathryn B. Muir, Clifford D. Packer

**Affiliations:** ^1^School of Medicine, Case Western Reserve University, Cleveland, OH 44106, USA; ^2^Louis Stokes Cleveland VA Medical Center, USA

## Abstract

Thrombocytopenia is a known potential side effect of hemodialysis, however, it is rarely seen in patients who undergo hemodialysis using biocompatible membranes. This case demonstrates hemodialysis-associated thrombocytopenia with use of biocompatible dialysis membranes that expose blood directly to polysulfone. The thrombocytopenia resolved in this patient when the dialysis membrane was changed to a biocompatible model with a polyethylene glycol barrier layer preventing direct interaction between patient blood and polysulfone. The calculated Naranjo ADR score of 9 indicates a highly probable adverse reaction.

## 1. Case

**Figure 1:**
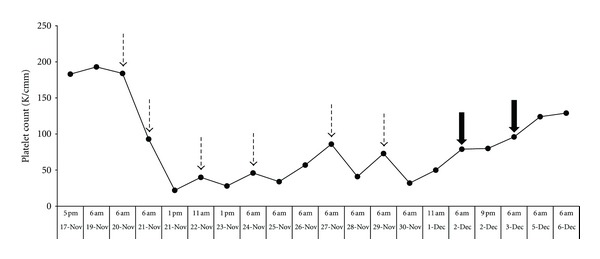
Demonstration of chronological platelet levels measured in this patient. Dashed arrows indicate administration of hemodialysis using either Optiflux 200 or 18NR polysulfone membrane dialyzers (Fresenius). Solid arrows indicate administration of hemodialysis using AM100 dialyzer with alkyl ether polymer-grafted cellulose membrane.

A 91-year-old African American man with a past medical history of chronic kidney disease presented with night sweats, myoclonic jerks, dysphagia, and loss of appetite all progressively worsening for approximately one year, in addition to a 40-pound weight loss over the past nine months. On admission, BUN and creatinine levels were 89 and 9.1, respectively. Hemodialysis was initiated on day four of hospital admission using the Fresenius Optiflux 200 dialyzer with a synthetic polymer (polysulfone) membrane. A nontunneled right internal jugular catheter was inserted for vascular access, and heparin was given per standard dialysis protocols. After two hemodialysis sessions over the course of 36 hours, the patient's platelet count decreased from 184,000 to 22,000. The hemoglobin and WBC remained at stable baseline levels of 10.4 g/dL and 4.13 × 10^9^/L, respectively, and continued in this range during the period of thrombocytopenia. Heparin-Induced Thrombocytopenia (HIT) was suspected; all heparin products and clopidogrel were discontinued and argatroban therapy was started. Despite this, the patient continued to demonstrate variably low platelet counts. A HIT panel, including PF4, PLT AB-Heparin dependent (SRA), and platelet antibody screen, was found to be completely negative and argatroban therapy was subsequently discontinued. With HIT no longer suspected, the patient was rechallenged with heparin during hemodialysis, with no change in the pattern of decreased platelet levels. On day thirteen of hospitalization, the dialyzer was switched to the Braun 18NR, also containing a synthetic polymer (alpha polysulfone) membrane; platelet levels continued to be variably depressed. Chronological correlation of platelet levels and hemodialysis sessions demonstrated a consistent decrease in platelet levels following each hemodialysis treatment (Figure 1) at which point it was suspected that thrombocytopenia was likely dialysis induced. The dialyzer was switched from the F18NR to the AM100 dialyzer with an alkyl ether polymer-grafted cellulose membrane. After initiating dialysis using the AM100, the patient's platelet levels began to recover without any regression. No episodes of postdialysis thrombocytopenia were recorded once the use of the AM100 was initiated. (See Table 1 for technical details of the 3 dialyzers used for this patient, and dates of use.)

## 2. Discussion

 Platelets have been known to interact with dialysis membranes since the 1970's; dialysis membranes have been shown to cause platelet adhesion, aggregation, and activation [1–5]. Platelet activation has been demonstrated by elevated levels of platelet factor 4 [2, 3] as well as thromboxane [4], following hemodialysis. Accordingly, thrombocytopenia is also a well-known complication of hemodialysis treatment. Hakim and Schafer suggested that thrombocytopenic episodes occurring with hemodialysis were associated with complement activation, specifically C3a, in addition to activation of platelets themselves [5]. Complement activation occurred specifically in the setting of cuprophane membranes, and thrombocytopenia was only observed in the presence of complement activation [4]. Verbeelen et al. showed that cellulose acetate dialyzer membranes can also cause transient thrombocytopenia and platelet activation [6]. In contrast, there were no changes in platelet levels and decreased complement activation when dialysis was undertaken using noncellulose polymethylmethacrylate membranes [5]. According to Verbeelen et al., polyacrylonitrile, hemophan, polysulfone, and cuprammonium membranes did not cause significant decreases in platelet levels in hemodialysis patients [6]. Thus, the use of biocompatible dialyzers has greatly reduced the occurrence of hemodialysis-associated thrombocytopenia. Recently, there have been three reported cases of thrombocytopenia likely induced by exposure to a biocompatible polysulfone dialyzer that resolved following the use of a hydrophilic gel-coated polysulfone dialyzer [7] or a cellulose triacetate dialyzer [8, 9]. In the case reported by Post in 2010 [7], the dialyzer in question was an Optiflux 160 (Fresenius Medical Care), which was subsequently switched to a REXEED 25S dialyzer with a hydrophilic gel layer coating the polysulfone membrane (Asahi Kasei Kuraray Medical). That patient recovered platelet levels but did continue to have recurrent episodes of thrombocytopenia associated with hemodialysis. In the case reported by Posadas et al. [8], hemodialysis-associated thrombocytopenia resolved when the patient was switched from a Fresenius F180NR Polysulfone dialyzer to a Baxter CT190G cellulose triacetate dialyzer. Olafiranye et al. [9] describe a similar case where thrombocytopenia resolved after switching from a Fresenius F200NR polysulfone synthetic dialyzer to a Baxter cellulose triacetate dialyzer.

 Kiaii et al. [10] have recently reported a cohort of 20 patients who developed thrombocytopenia following electron beam sterilization of polysulfone dialysis membranes. The thrombocytopenia resolved when nonelectron beam sterilization methods were used. The authors postulate that electron beam radiation might affect membrane integrity, structure, or physical properties that could lead to platelet activation, aggregation, or adsorption, causing thrombocytopenia. In our patient's case, electron beam sterilization was used initially with the Optiflux 200 dialysis membrane, but the thrombocytopenia recurred when he was switched to a Braun 18NR dialyzer that was sterilized with gamma radiation. It therefore appears unlikely that electron beam membrane irradiation was the sole cause of thrombocytopenia in our patient. 

 The notable difference between the Fresenius membranes and the AM100 membrane used in this patient, all of which were polysulfone membranes, is the presence of a thin polyethylene glycol (alkyl ether) layer covering the polysulfone membrane in the AM100. According to Ashai, the manufacturer, the AM100 is made of modified cellulose (alkyl ether polymer-grafted cellulose) derived from cuprammonium rayon. The cuprammonium rayon is manufactured to have a thin layer of modified cellulose exposed to blood contract surfaces, making it more biocompatible as manifested by lower complement activation (C3a and C5a) compared to regular cellulose. Neither of the first two dialyzers used on our patient contained a polyethylene glycol layer. The presence of this layer in the AM100 membrane prevents direct interaction between polysulfone and platelets during hemodialysis, which may be responsible for prevention of platelet activation. The cellulose triacetate dialysis membranes mentioned above [8, 9] may also reduce platelet aggregation membranes by the virtue of their decreased activation of bound glycoprotein IIb/IIIa as compared with polysulfone membranes [11].

 The exact mechanism of thrombocytopenia in our patient is unknown. Perhaps contact between a variant platelet surface protein and polysulfone resulted in platelet activation or agglutination and thrombocytopenia. Perhaps this patient was particularly susceptible to C3a activation following interaction with polysulfone, indirectly causing platelet activation and thrombocytopenia. It is uncommon for a 91-year-old to be initiated on dialysis, and age may have played a role in this patient. In older patients, there is a higher likelihood of point mutations in platelet precursors, which could theoretically produce platelets more susceptible to activation, resulting in thrombocytopenia.

 This case of hemodialysis-associated thrombocytopenia in a new dialysis patient demonstrates that polysulfone dialysis membranes can variably affect platelet levels, despite previous evidence indicating that polysulfone membranes do not affect platelet counts [6]. Based on the Naranjo ADR Probability Scale [12] score of 9, it is highly probable that the observed episodes of thrombocytopenia were the result of hemodialysis using two different polysulfone dialysis membranes. Explanation of the Naranjo score is as the following.

 (+1) *There are previous conclusive reports on this reaction* (see [7–9]).
 (+2) *The adverse event appeared after the suspected drug was administered. *
  (+1) *The adverse reaction improved when the drug was discontinued. *
  (+2) *The adverse reaction reappeared when the drug was readministered. *
  (+2) *There were no clear alternate causes (other than the drug) that could have caused the adverse reaction.* HIT was ruled out, and no other drugs or local factors were implicated. Electron beam sterilization was not a likely cause since the patient also developed thrombocytopenia from a gamma-irradiated polysulfone dialysis membrane (the Braun Diacap 18NR).
  (+1) *The patient had a similar reaction to the same or similar drug.* The patient developed similar degrees of thrombocytopenia with two different polysulfone dialysis membranes.
 Total Score: (+9).

Hemodialysis-associated thrombocytopenia is an important, if rare, potential complication of hemodialysis that should be assessed via monitoring of platelet levels in both patients newly initiated on hemodialysis therapy as well as those with recent modifications in their hemodialysis regimens. This case demonstrates that there may be a small subset of patients for whom direct contact of platelets with polysulfone results in thrombocytopenia. This reaction may be preventable with use of a dialysis membrane that is treated with polyethylene glycol to eliminate a direct interaction between platelets and polysulfone. Cellulose triacetate dialyzers may also be a good option for these patients.

## Figures and Tables

**Table 1:** Details on the 3 dialyzers used between 11/20/10 and 12/7/11.

Name: Optiflux 200
Manufacturer: Fresenius
Size: 2.0 square meter surface area
Membrane: polysulfone
Sterilization: electron beam
Dates used: 11/20, 11/21, 11/22, 11/24, 11/27

Name: Diacap 18NR
Manufacturer: B Braun
Size: 1.8 square meter surface area
Membrane: alpha polysulfone
Sterilization: gamma irradiation
Dates used: 11/29

Name: AM-BIO-100
Manufacturer: Asahi
Size: 2.0 square meter surface area
Membrane: alkyl ether polymer-grafted cellulose
Sterilization: gamma irradiation
Dates used: 12/2, 12/4, 12/7
